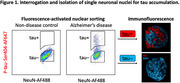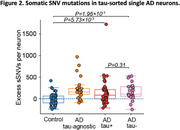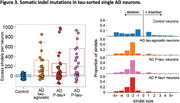# Neurons accumulate disease‐specific somatic deletions across tau pathological states in Alzheimer’s disease

**DOI:** 10.1002/alz70861_108747

**Published:** 2025-12-23

**Authors:** Michael B Miller, Bowen Jin, Katherine Brown, Denis Smirnov, August Yue Huang

**Affiliations:** ^1^ Brigham and Women's Hospital, Boston, MA USA; ^2^ Boston Children's Hospital, Boston, MA USA

## Abstract

**Background:**

Tau deposition within neurons marks Alzheimer’s disease (AD) neuropathology, suggesting that tau may directly drive cellular dysfunction and death. In advanced AD, somatic mutations accumulate in neuronal genomes, with features that suggest deleterious effects on cellular function.

**Method:**

To examine the relationship between tau and somatic mutation, we isolated single neurons according to tau pathology (Figure 1) and performed single‐cell whole‐genome sequencing on tau+, tau–, and tau‐agnostic neurons from 7 individuals with AD, as well as neurons from 15 control individuals.

**Result:**

We found that AD neurons, regardless of their tau status, exhibited an increased burden of somatic single‐nucleotide variants (sSNVs, Figure 2) and insertions and deletions (sIndels, Figure 3). Mutational signature analyses implicate disease‐associated oxidative damage as a contributor to sSNVs and reveal a novel disease‐specific sIndel pattern characterized by two‐basepair deletions, indicating shared mutagenic mechanisms on AD neurons across tau pathological cell states. In contrast to these single‐neuron findings, immunohistochemistry showed that somatic mutations are associated with tissue‐wide tau pathology.

**Conclusion:**

These findings suggest that tangles do not confer cell‐autonomous genotoxicity to neurons and that non‐tangle components drive somatic mutation in AD.